# Beyond passive observation: feedback anticipation and observation activate the mirror system in virtual finger movement control via P300-BCI

**DOI:** 10.3389/fnhum.2023.1180056

**Published:** 2023-05-04

**Authors:** Nikolay Syrov, Lev Yakovlev, Andrei Miroshnikov, Alexander Kaplan

**Affiliations:** ^1^V. Zelman Center for Neurobiology and Brain Rehabilitation, Skolkovo Institute of Science and Technology, Moscow, Russia; ^2^Baltic Center for Neurotechnology and Artificial Intelligence, Immanuel Kant Baltic Federal University, Kaliningrad, Russia; ^3^Department of Human and Animal Physiology, Faculty of Biology, Lomonosov Moscow State University, Moscow, Russia

**Keywords:** action observation (AO), mirror neurons, brain–computer interface (BCI), error-related potentials (ErrPs), sensorimotor cortex, feedback anticipation, beta synchronization

## Abstract

Action observation (AO) is widely used as a post-stroke therapy to activate sensorimotor circuits through the mirror neuron system. However, passive observation is often considered to be less effective and less interactive than goal-directed movement observation, leading to the suggestion that observation of goal-directed actions may have stronger therapeutic potential, as goal-directed AO has been shown to activate mechanisms for monitoring action errors. Some studies have also suggested the use of AO as a form of Brain–computer interface (BCI) feedback. In this study, we investigated the potential for observation of virtual hand movements within a P300-based BCI as a feedback system to activate the mirror neuron system. We also explored the role of feedback anticipation and estimation mechanisms during movement observation. Twenty healthy subjects participated in the study. We analyzed event-related desynchronization and synchronization (ERD/S) of sensorimotor EEG rhythms and Error-related potentials (ErrPs) during observation of virtual hand finger flexion presented as feedback in the P300-BCI loop and compared the dynamics of ERD/S and ErrPs during observation of correct feedback and errors. We also analyzed these EEG markers during passive AO under two conditions: when subjects anticipated the action demonstration and when the action was unexpected. A pre-action mu-ERD was found both before passive AO and during action anticipation within the BCI loop. Furthermore, a significant increase in beta-ERS was found during AO within incorrect BCI feedback trials. We suggest that the BCI feedback may exaggerate the passive-AO effect, as it engages feedback anticipation and estimation mechanisms as well as movement error monitoring simultaneously. The results of this study provide insights into the potential of P300-BCI with AO-feedback as a tool for neurorehabilitation.

## 1. Introduction

Brain–computer interfaces (BCIs) offer a methodological and technological paradigm that allows individuals to control external devices via mental commands without the need for physical activity (Wolpaw, 2007). The detection of mental commands is achieved through the measurement and interpretation of brain activity recorded via electroencephalography (EEG) and other methods. BCIs have become widely used in rehabilitative medicine, serving as communicators, and neurorehabilitation tools that offer therapeutic benefits for motor recovery (Bamdad et al., 2015; Mrachacz-Kersting et al., 2021).

P300-based BCIs, along with other event-related potential (ERP)-based paradigms, are frequently employed in clinical settings to assist individuals with severe disabilities to communicate or interact with their environment (Bamdad et al., 2015; Delijorge et al., 2020). These BCIs present the user with external stimuli and measure the brain’s response using EEG to determine which stimulus the user was paying the most attention to.

On the other hand, motor-imagery-(MI)-based BCIs detect changes in brain activity that occur when a user imagines performing a specific action (Neuper et al., 2006). The brain areas activated during MI overlap with the sensorimotor networks involved in actual movement preparation and execution (Pfurtscheller and Neuper, 1997; Pfurtscheller, 2000). Therefore, MI-based BCIs serve in motor rehabilitation and help patients with neurological disorders or injuries to improve their motor skills and regain movement control (Khan et al., 2020).

However, to achieve acceptable accuracy in MI-based BCI control, proper training of kinesthetic imagery skills is needed, which may take time to learn. Moreover, MI-based BCIs cannot be used by many post-stroke patients because they may have an impaired ability to generate a vivid kinesthetic motor image (Sirigu et al., 1996; Malouin et al., 2007; Hougaard et al., 2022). Insufficient BCI accuracy leads to a lot of error feedback, which can cause patients to lose control of the BCI, feel frustrated, and lose motivation. This loss of motivation can play a crucial role in the rehabilitation outcome (Fard and Grosse-Wentrup, 2014; Hougaard et al., 2021, 2022). The feedback signals resulting from the user’s intentions have the capacity to potentiate Hebbian-associative neuroplasticity, which reinforces the networks responsible for successful behavioral responses or behavioral changes in the case of failure (Grosse-Wentrup et al., 2011). Therefore, it is essential to provide appropriate feedback in rehabilitative systems based on BCI technology.

In contrast to MI-BCIs, the P300-based paradigm has several significant advantages. Research has demonstrated that P300 can be detected in the electroencephalography (EEG) of post-stroke individuals with an accuracy rate of approximately 70–80%, which is lower than the accuracy achieved by non-disabled individuals but still sufficient for successful BCI control (Guger et al., 2009; Ortner et al., 2011; Anitha et al., 2019). Additionally, P300 BCIs allow users to choose a target stimulus by switching attention to that stimulus, so the number of possible BCI commands depends only on the system design. With the advantage that MI-BCIs do not require an external stimulus environment, they offer a smaller number of commands and are typically limited to three or four recognizable motor images (Wang et al., 2012; Pei et al., 2021).

All of these factors make the P300 paradigm an attractive tool for creating a closed-loop BCI-based rehabilitation system with high reliability in realizing the patient’s intentions. However, P300-based BCIs have only recently been considered as a tool for motor rehabilitation (Kaplan et al., 2016). So, Delijorge et al. (2020) proposed using the P300-based BCI to control a hand exoskeleton, which allows users to control all exoskeleton fingers with high accuracy, while MI-based paradigms are limited in their ability to provide fine movements of finger control. Several studies have proposed combining exoskeletons, virtual reality, and electrical stimulation to restore both upper limb (Bulanov et al., 2021; Hernandez-Rojas et al., 2022) and lower limb (Ninenko et al., 2022) mobility in individuals with paralysis, using the P300-BCI to control the robotic-manipulator and electrical stimulation.

In a previous study, we demonstrated that observation of virtual hand movements in a BCI-P300 loop resulted in increased corticospinal excitability compared to passive observation of the same movements (Syrov et al., 2019). Our approach aims to employ P300-BCI to enable paralyzed patients to control the movements of a virtual avatar. Action observation has been widely used in post-stroke therapy to activate sensorimotor circuits via the mirror neuron system (Ertelt et al., 2007). We propose that observation of movements within the BCI feedback could enhance the passive AO effects by engaging feedback anticipation and estimation brain mechanisms, as well as movement error monitoring. Our suggestion is that mirror neurons may play a role in BCI-feedback processing by comparing the observed action with the intended movement and detecting discrepancies between them, which could activate areas involved in motor-error monitoring (Bates et al., 2005; Pyasik et al., 2022). In EEG studies, activation of sensorimotor areas during action observation has been observed via the evaluation of mu and beta rhythm event-related desynchronization (ERD), which indicates a relative decrease in amplitude of oscillatory activity (Frenkel-Toledo et al., 2014; Lapenta et al., 2018).

Moreover, beta synchronization (beta ERS) was detected during the observation of incorrect movements (Koelewijn et al., 2008). Beta ERS has been proposed as a marker of primary motor cortex inhibition, which suggests that the observation of motor errors triggers M1 deactivation as a mechanism for correcting mirrored movements. In our study, we aimed to investigate the amplitude of sensorimotor EEG activity during the observation of virtual hand movements presented as feedback in the P300-BCI loop. We compared the dynamics of mu and beta ERD and beta ERS during the observation of correct feedback and errors. In addition, we explored error-related potentials (ErrPs) to confirm that error monitoring occurs.

## 2. Materials and methods

### 2.1. Participants

We recruited 20 healthy adult right handed participants (age range: 25 ± 3 years-old; 12 females). All participants reported no history of neurological or psychiatric disorders, and were not taking any medication that could affect the central nervous system. Participants provided written informed consent prior to participating in the study. The experimental protocol was approved by the Lomonosov Moscow State University Committee for Bioethics (protocol no. 111-ch) and was conducted in accordance with the Declaration of Helsinki Ethical Principles for Medical Research Involving Human Subjects.

### 2.2. Experimental procedure

The individuals taking part in the study were seated comfortably in an armchair. They were instructed to keep their hands relaxed while performing two tasks involving visual stimuli displayed on an 18-inch LCD screen located 50 cm in front of them. Prior to the experimental session, the participants were asked to perform a brief set of voluntary finger movements. For this task, the subject was shown the name of the finger to move on the monitor screen, followed by a command to move it after a second. During the task, both EEG and EMG were recorded. The purpose of this task was to evaluate the individual spatial-frequency characteristics of the sensorimotor rhythms ERD/S.

#### 2.2.1. BCI action observation task (BCI-AO)

**FIGURE 1:**
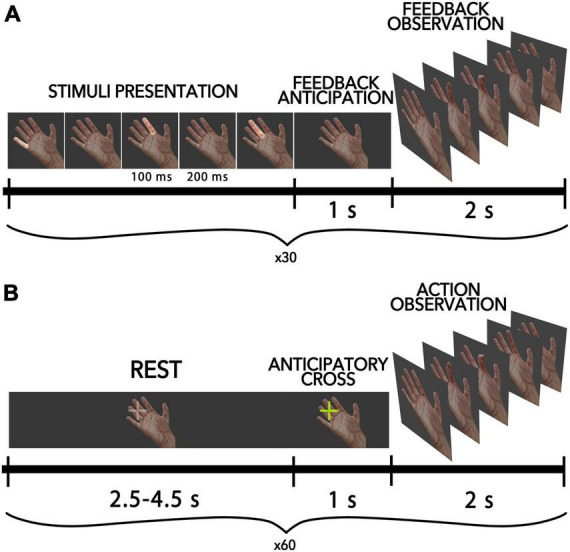
Schematic diagram of the experimental pipeline for all conditions. **(A)** AO-BCI condition, where participants controlled virtual finger flexions with P300-BCI. Each trial includes the presentation of visual stimuli, a period of anticipation, and the presentation of feedback in the form of a video sequence with finger movements. **(B)** Passive AO condition, where half of all trials included an anticipatory cross-presentation 1 s before movement onset to warn participants of the action.

The present study employed a BCI-AO task utilizing the P300-based paradigm, whereby finger movements of the virtual anthropomorphic hand were presented on the screen as feedback within the BCI loop. Figure 1A shows the scheme of this condition. A virtual hand was displayed on a screen located in front of each participant (an 18.5-inch LCD monitor was placed in front of the subject at 50 cm from their eyes). During each trial, participants were tasked with inducing finger flexion via mental commands transmitted through the BCI. Each participant completed a total of 40 trials. In accordance with the P300-based BCI paradigm, within each trial, all fingers were sequentially highlighted in a random order, with each finger being highlighted 10 times for a total of 40 flashes per trial. Each highlight lasted for a duration of 100 ms, with a 200 ms interstimulus interval. To elicit the movement of a particular virtual finger (i.e., the target finger), participants were instructed to selectively attend to the highlighting of that finger: they were asked to mentally count the number of target flashes to help them focus their attention. When non-target fingers were highlighted, participants were not required to take any action, so non-target flashes were ignored. The EEG responses elicited in response to finger highlights were utilized to determine the direction of attentional focus and initiate the flexion of the target finger. A linear discriminant classifier from the SciKit-Learn Python library v0.23.2 (Pedregosa et al., 2011) was utilized with default settings. To provide a sufficient number of erroneous feedback, we included a random component in the decision algorithm that randomly chose a finger to move differently from what was chosen by the participant. Such errors occurred in 15 cases out of the total attempts, resulting in an average accuracy of not more than 62.5%. The participants weren’t aware of this invasion.

#### 2.2.2. Passive action observation task

In the passive action observation condition, participants observed intransitive finger movements identical to those in the BCI feedback, except that they were presented outside of the BCI loop. The stimuli lasted for 2 s, with 1 s for finger flexion and 1 s for finger extension. The virtual finger movements were presented to participants in a pseudorandom sequence. During the intervals between finger movements, a resting hand was displayed on the screen, with a gray cross positioned before the fingers to fixate the participant’s gaze in that field. The duration of the resting period varied randomly from 2.5 to 4.5 s. In total, 60 actions were demonstrated to a participant. The fixation cross changed its color to yellow (“anticipatory cross”) in half of the cases 1 s before the movement onset, signaling the start of the finger movement to the participant (see Figure 1B). This created two types of conditions in the passive AO task: the anticipated action observation condition (referred to as “AAO”), in which participants were warned about the hand movement in a random order during 50% of trials, and the “AO” condition, in which the finger movement was launched unexpectedly for the participant (the cross did not change color).

### 2.3. EEG recording and preprocessing

The EEG was recorded using an NVX52 DC amplifier (MKS, Russia) with 48 Ag/AgCl scalp electrodes, placed according to the “10–10” international system in the following positions: *Fp1, Fp2, F5, F3, F1, F2, F4, F6, FT7, FT8, FC5, FC3, FC1, FCz, FC2, FC4, FC6, C5, C3, C1, Cz, C2, C4, C6, CP5, CP3, CP1, CPz, CP2, CP4, CP6, T7, T8, P7, P5, P3, P1, Pz, P2, P4, P6, P8, PO3, POz, PO4, O1, Oz*, and *O2*. The contact resistance for each of the electrodes was kept below 20 kΩ. The signal was sampled at 500 Hz with a 50 Hz notch filter.

The EMG signal was also recorded synchronously from the flexor digitorum superficialis muscle (FDS) and extensor digitorum communis muscle (EDC) of the right hand to check muscle activity.

The EEG data was preprocessed by first visually inspecting the raw signal to remove epochs with poor signal quality. The signal was then filtered using a 4th order Butterworth filter with a frequency range of 1–40 Hz. Independent component analysis (ICA) was applied to identify and remove eye blink and muscle artifacts from the EEG data. Frontally localized ICA components with high correlation with Fp1 and Fp2 channels were classified as EOG artifacts, while those distributed on T7-T8 channels were classified as muscular artifacts. Finally, the EEG data was re-referenced to the average reference. The MNE-Python toolbox was utilized to perform these preprocessing steps (Gramfort et al., 2013).

#### 2.3.1. Event-related potentials processing

Event-related potential analysis was performed to assess error-related processing (correct vs. erroneous actions) in the AO BCI condition and in the passive action observation condition to estimate differences in action-related potentials in the AAO and AO trials. EEG signal was epoched into [−1; 1] s intervals locked to the movement onset, mean value in [−1; −0.2] s interval was used for baseline correction. Epochs were then averaged for each subject according to condition.

Error-related potentials were statistically compared with EEG responses from trials with correct feedback. To avoid multiple comparisons, we used a non-parametric spatio-temporal cluster-based test with a threshold of 20 and 10,000 permutations (Maris and Oostenveld, 2007). The significance level was set at 0.05.

#### 2.3.2. Analysis of sensorimotor rhythms (de-)synchronization

##### 2.3.2.1. Common spatial pattern algorithm for ERD/S sources extraction

To ensure the sensorimotor specificity of the analyzed ERD/S responses related to action observation and to eliminate contamination from occipital alpha activity, we used the common spatial pattern (CSP) algorithm. The CSP algorithm is a commonly used method for extracting features related to motor imagery from multi-channel EEG data (Rithwik et al., 2022). It involves a linear transformation of the multi-channel EEG data from two different conditions into a low-dimensional common spatial subspace, achieved by a set of spatial filters that maximize the variance of two-class signal matrices. In our study, these two classes were defined as the “active state,” i.e., action observation (or BCI feedback observation), and the “resting state,” which involved observation of a motionless hand. We have successfully used CSP for the extraction of AO-related changes in sensorimotor EEG (Syrov et al., 2022). In order to create sensitive spatial filters for ERD/S analysis, we determined subject-specific frequency subranges that corresponded to the mu (6–15 Hz) and beta (12–30 Hz) frequency ranges where the ERD and ERS occurred. These subranges were determined through visual inspection of time-frequency perturbations in action execution trials.

The EEG data was then filtered according to these subranges. The data were then divided into epochs corresponding to “active state” (2 s from 0 to 2 s after movement onset) and “resting state” (0.5–2.5 s after movement onset for the passive AO condition and 4–2 s before feedback presentation for the AO-BCI condition). The anticipation period was not used in the CSP fitting for both the AAO and AO-BCI conditions. The implementation of the CSP algorithm was taken from the Python library MNE 0.23 (Gramfort et al., 2013) with modifications [a procedure for cleaning the covariance matrix by removing unrepresentative “noisy” epochs was added, as suggested in Cohen (2022)].

**FIGURE 2:**
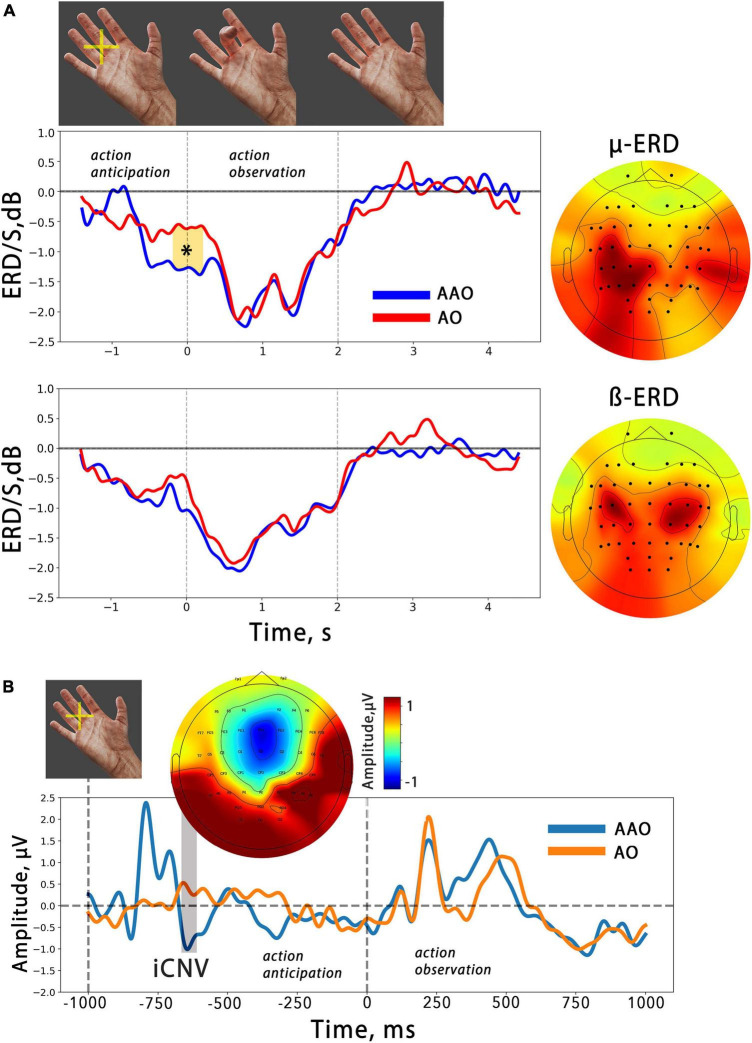
**(A)** The averaged temporal dynamics of mu/beta ERD during action anticipation and observation in AAO (blue line) and AO (red line) conditions. Colored shapes with asterisks indicate significant differences. The vertical lines delimit the 2-second time intervals during which a finger movement was presented. On the right, the averaged spatial patterns corresponding to each EEG reaction are displayed. **(B)** Averaged evoked potentials related to action observation in two different conditions. The ERP evoked by anticipatory cross occurred before action presentation and is marked as “iCNV”. Above the topography distribution of its amplitude is shown. The first dashed line indicates the moment of anticipatory cross presentation, while the second indicates the moment of finger flexion onset.

**FIGURE 3:**
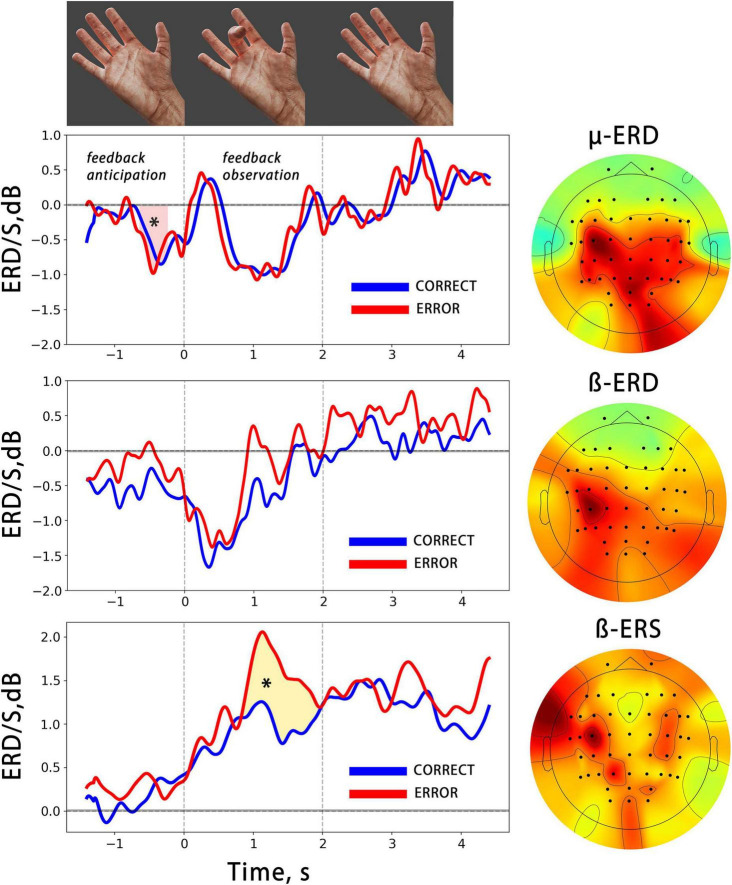
The averaged temporal dynamics of mu/beta ERD and beta ERS (bottom) during anticipation and observation of actions presented as BCI feedback. The blue line denotes observation of CORRECT actions, while the red line denotes observation of ERRONEOUS actions colored shapes with asterisks indicate significant differences. The vertical lines limit the 2 s time intervals during which the BCI feedback was presented. On the right, the averaged spatial patterns corresponding to each EEG reaction are shown.

Spatial filters with only central (over the sensorimotor areas) localization of spatial patterns were taken for further analysis. They were applied to the broadly filtered data obtained in the pre-processing step. This procedure transformed the raw data into components that could be interpreted as a complex mixture of signals from the original EEG data, including specific components of a target mental state [see Arvaneh et al. (2011) and Kim Y. et al. (2016) for more details]. The selected spatial patterns were then averaged and visualized (see Figures 2, 3). The spatial pattern visualization helps to understand the topographic distribution of the features extracted from the raw EEG signal that are most informative for distinguishing between the two different states, i.e., action observation and REST.

##### 2.3.2.2. Time-frequency analysis and ERD/S evaluation

For the time-frequency analysis, we utilized the Morlet wavelet transform, using a set of complex Morlet wavelets with a variable number of cycles for different frequencies. The frequencies of the wavelets ranged from 3 to 30 Hz in steps of 0.3 Hz. The full width at half maximum (FWHM) was set to 140 ms, which corresponds to a spectral FWHM of 4.5 Hz. To calculate the desynchronization value, the signal power in the action observation conditions was divided by the median of the signal power in the resting state (median was taken over all trials and time stamps). The resulting values were then converted to decibels, with negative values corresponding to ERD and positive values corresponding to ERS. The temporal dynamics of ERD/S were obtained by taking the median across all epochs and within subject-specific frequency subranges.

For statistical analysis, we used a non-parametric cluster-based test with threshold-free cluster enhancement and 10,000 permutations. To identify significant differences in ERD during the feedback anticipation period, we compared the temporal dynamics of mu/beta desynchronization with the zero-level corresponding to the resting state. We also compared ERD/S dynamics between incorrect BCI output trials and correct feedback trials. In addition, we compared mu/beta ERD dynamics between AO and AAO conditions to explore the effects of action monitoring awareness.

## 3. Results

**FIGURE 4:**
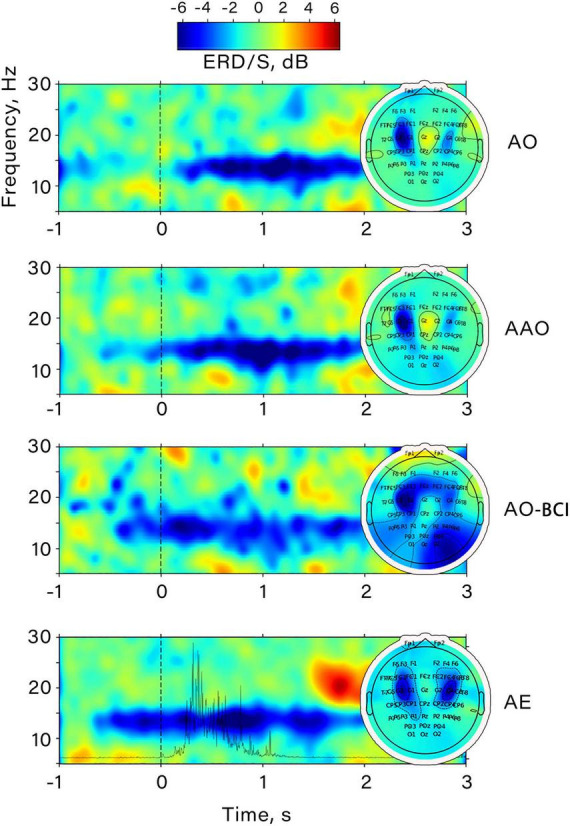
Example of the time-frequency dynamics of the ERD/S value obtained prior to CSP application for a participant. The data corresponds to channel “C3”. All conditions are shown: AO, passive action observation; AAO, passive action observation with action anticipation; AO-BCI, observation of actions presented as BCI feedback; AE, action execution. The spatial distribution of ERD/S for each condition is shown on the right.

Figure 4 shows an example of the time-frequency dynamics for one subject. It is notable that both action anticipation in passive action observation and within BCI feedback perception induce desynchronization in the mu range, which increases further during the observation of a finger movement. A similar pattern of event-related desynchronization (ERD) was also discovered during voluntary finger flexions, as ERD appeared in the same frequency range. It’s noteworthy that the amplitude decrease occurred almost 1 s before the movement onset, and its maximum corresponded with the moment of maximum EMG activity power. The central distribution of the most pronounced desynchronization in all conditions suggests that the mu rhythm was elicited during action observation and BCI feedback observation, indicating the activation of sensorimotor regions.

An analogous spatial pattern in the mu ERD distribution was also revealed in the averaged CSP patterns. Figure 2A displays the averaged mu/beta ERD CSP-sources and the average dynamics of the spectral power in these frequency bands, which were obtained using selected spatial filters.

It is noteworthy that the mu ERD spatial pattern is skewed toward the parietal area, while the beta ERD pattern demonstrates bilateral fronto-central localization. The analysis of the desynchronization dynamics revealed a gradual decrease in both mu and beta oscillations during action observation. A cluster-based permutation test was performed, which found one temporal cluster starting just before the action onset. Within this time interval, the test indicated a significantly greater ERD in the mu range related to virtual finger action anticipation. However, no such effects were found in the beta band. Summarized results of the statistical tests applied are presented in Table 1.

**TABLE 1:** Results of statistical tests.

ERD/S in action anticipation			
Frequency range	Cluster’s time range, s	Mean F	*p*-Value
mu-ERD	−0.2 to 0.25	11.38	0.005
**ERD/S in BCI feedback**			
mu-ERD	−0.7 to 0.0	14.84	0.001
beta-ERS	0.8 to 1.75	4.1	0.04
**oErrP in BCI feedback**			
**Cluster’s spatial points**	**Cluster’s time range, s**	**Mean F**	***p*-Value**
F1, FC1, C1, FCz, Cz, FC2	0.470 to 0.520	9.3	0.0002

Figure 2B shows the average ERPs locked to the onset of finger movement in two action observation conditions. The ERPs consist of several peaks, and it can be observed that the early potentials are similar in simple AO and anticipated (AAO), but the late positive potential (in the 400–600 ms time range) in the AO condition begins with a slight delay compared to the AAO. However, the results of the cluster-based permutation test did not reveal any significant differences between the two conditions.

We analyzed the time course of mu/beta ERD during the BCI control, and found a significant decrease in the mu rhythm, but not in the beta rhythm, amplitude during the feedback anticipation. A cluster-based permutation test revealed a significant difference between the pre-action mu ERD and zero value (*p* = 0.005, mean F-score = 11.38, see Figure 3). Both the mu and beta oscillations decreased during the action observation, which was presented as BCI feedback. It is important to note that the correctness of the presented feedback (correct vs. error) did not have an effect on either the mu or beta ERD. We also analyzed the synchronization reaction in the low-beta range (average frequency range of beta ERS for all participants is 12–17 Hz) and found that it reached significantly higher values during error observation, i.e., observation of wrong fingers, which differed from what the participant tried to choose with the BCI (see Figure 3).

It’s important to note that the chosen spatial filters applied to EEG had central localization of the spatial patterns. The average weighted CSP patterns for mu/beta ERD and beta ERS reactions are shown in Figure 3. They display a predominantly contralateral distribution of the CSP features, which maximizes the variance of EEG signals in target frequency ranges between action observation and “rest” conditions.

**FIGURE 5:**
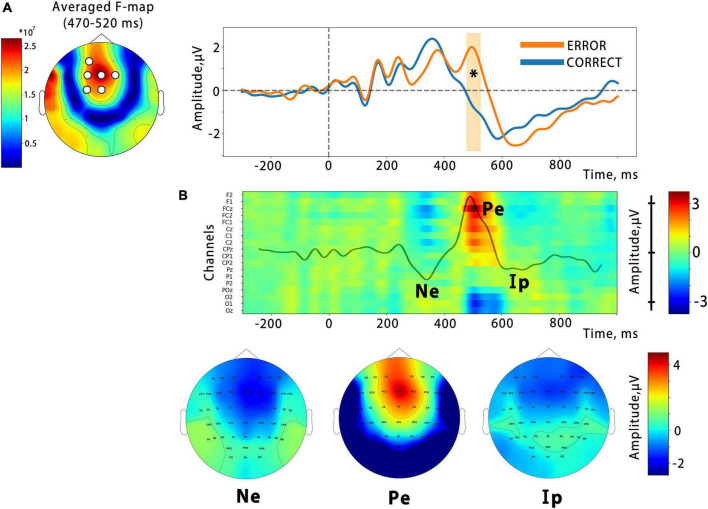
**(A)** Significant differences from the cluster-based permutation test. On the left is the F-score map, with significant channels highlighted. The average ERPs waveform for the spatial cluster is shown on the right, with the time interval containing significant differences highlighted (*p* = 0.0002). The vertical line indicates the onset of the finger action. Panel **(B)** is a color map that depicts the temporal evolution of the amplitude of the difference ErrP (Error–Correct) at the midline and nearest electrodes. Specific ErrP components are denoted: Ne, error-related negativity; Pe, error-related positivity; Ip, interaction potential. Corresponding topographic maps are presented below.

In order to examine the temporal dynamics of the error feedback processing, we conducted an event-related potential (ERP) analysis. Our analysis revealed that the ERP response triggered by the virtual hand action presented as BCI feedback consisted of several peaks that differed between correct and error outputs in the time ranges of 200–300 ms (corresponding to the error-related negativity, Ne), 450–600 ms (error-related positivity, Pe), and the latest deflection with a latency of around 700 ms, which corresponds to the interaction potential (Ip). A spatio-temporal permutation test showed a significant difference between the ERP curves in a temporal cluster that coincided with the Pe latency (cluster from around 470 to 520 ms with the mean F-score = 9.3, *p* = 0.0002). The cluster was located at central F, FC, and C electrode sites, as can be seen in Figure 5, which presents an F-score map with highlighted significant channels. This is consistent with the resulting fronto-central topographic distribution for the Pe peak amplitude (see Figure 5B). The calculation of the effect size for the identified cluster, based on the data extracted from the channels and time interval corresponding to the cluster, revealed a significant effect with a large magnitude (Cohen’s *d* = 1.23).

As shown in Figure 5B, the error-related negative potential and the interactive potential were more negative during the observation of incorrect actions, but neither reached significance.

## 4. Discussion

The study of feedback anticipation and estimation is a crucial aspect in the realm of brain-computer interaction. The examination of error-related (feedback-related) potentials is a widely-used approach to gain insight into the neural basis of these processes. In this study, our focus was on examining the changes in sensorimotor EEG activity during observation of BCI feedback that was in the form of an anthropomorphic palm finger. It is well-established in the literature that the mu and beta ERD/S over sensorimotor cortical areas decrease during action observation and are sensitive to action characteristics and correctness. However, there are few published findings to date on the ERD/S during the process of anticipation of an upcoming action and estimation of an action presented within BCI feedback processing.

### 4.1. Mu and beta desynchronization during action anticipation and observation

Our results align with previous studies that have shown mu/beta event-related desynchronization during both action execution and observation, suggesting that cortical networks involved in mu/beta sensorimotor activity are part of the mirror neuron system or the action observation network (van Schie et al., 2008; Lapenta et al., 2018). Both mu and beta activity exhibit a decrease in amplitude that occurs synchronously with the observed finger flexion: ERD increases during flexion, reaching a maximum value at the moment of maximal flexion aperture, before returning to baseline during the extension phase. This result aligns with previous findings (Avanzini et al., 2012; Syrov et al., 2022). The study also revealed a differential development of mu and beta ERD during the anticipation phase of actions, suggesting distinct roles of these rhythms in action anticipation and supporting the notion that mu and beta ERD are sensitive to different aspects of action “mirroring” (Shibuya et al., 2021; Syrov et al., 2022). Specifically, in our study, we observed a significant mu ERD during the anticipatory phase prior to action onset, while beta ERD was not observed (Figure 2).

Figure 4 allows a comparison of the temporal dynamics of the ERD during both actual action execution and action observation. The amplitude of the mu rhythm decreases about 1 s before action onset, indicating preparation processes in the sensorimotor circuitry (Pfurtscheller, 2000). Compared to preparation for action execution, preparation for action observation is less frequently described in the literature and is mostly studied in the context of goal-directed action trajectory prediction (Lago and Fernandez-del-Olmo, 2011). Studies have shown that general activation of M1 appearing before action observation reflects muscle-non-specific action prediction and is associated with predicting the goal of the object-directed movement rather than the resonance of specific muscle activation (Lago and Fernandez-del-Olmo, 2011; Naish et al., 2014). And the subsequent action observation activates M1 in a muscle-specific manner (Naish et al., 2014).

Studies investigating anticipation for action observation using EEG are relatively scarce in the literature. Kilner et al. (2004) found that a slow readiness potential developed both before execution and observation of an action, suggesting that knowing about an upcoming movement is enough to activate one’s own motor system, allowing people to anticipate the actions of others. However, the nature of the readiness potential is debated and might not be related to specific movement preparation processes but rather represents an averaging of random spontaneous fluctuations in neural activity while waiting for any event (Schurger et al., 2012). The late components of contingent negative variation (CNV) represent both motor preparation and stimulus anticipation, according to the authors (Brunia and Damen, 1988). CNV is elicited after the anticipatory stimulus but before the imperative stimulus, which engages the participant to execute an action. The CNV is typically elicited after an anticipatory stimulus before an imperative one that engages the participant to execute an action (Glazer et al., 2018). In the present study, an anticipatory cross flash was used to engage participants in waiting for an action, similar to the anticipatory stimulus in the CNV paradigm. Thereby, the ERP obtained in response to the anticipatory cross in this study may be comparable to the CNV. Figure 2B shows the obtained ERP, with the early component peaking at the FCz channel resembling the early CNV component (iCNV) associated with an orienting response, while the late phase reflects stimulus anticipation and/or response preparation (Bender et al., 2004; Röhricht et al., 2018).

However this late phase is not distinguishable in our results due to the short time interval between the anticipatory and imperative stimuli (Röhricht et al., 2018). Nevertheless, significant mu ERD was observed, indicating the activation of somatosensory areas during anticipation of action observation.

These findings are consistent with those of an fMRI study (Abreu et al., 2012), in which S1 activation was observed during action anticipation. It has been suggested that the preparation of upcoming actions involves a motor simulation that maps specific sensory features of the action (Bernstein, 1947; Debnath et al., 2019). This process is likely carried out via the somatosensory cortex, as supported by literature data (Caetano et al., 2007). Additionally, the same brain areas might be involved in the anticipation of others’ actions, as evidenced by our results and previous studies.

It can be noted (Figure 2) that a slight pre-action ERD also occurred in the AO condition, where the finger flexion started suddenly. We can explain this by considering that the interval we used between two subsequent movements in the AO condition was short and predictable, which may have caused the anticipation effect in the passive observation condition. Notably, this effect was insignificant and the anticipation ERD was significantly larger in the AOA condition compared to AO.

Surprisingly, beta ERD didn’t change during the anticipation of AO. As beta ERD is suggested as an M1 activation marker, we expected a decrease in beta amplitude that would be matched with TMS studies (Lago and Fernandez-del-Olmo, 2011; Naish et al., 2014). The distinguishing role of mu and beta rhythms in action prediction is hypothesized here. The study (Monroy et al., 2019) investigated the relationship between statistical regularities and motor predictions during action observation using (Ghilardi et al., 2023) showed that the mu rhythm is generally related to action prediction, independent of the statistical structure of the action. However, the beta rhythm appears to be sensitive to action probability, with beta ERD being higher during anticipation and observation of more probable actions. In our study, the finger to be flexed was selected at random with equal probability for each finger, which may explain the lack of beta ERD during the anticipation period (Ghilardi et al., 2023).

### 4.2. Mu and beta desynchronization during the BCI-feedback anticipation and observation

Our study demonstrated that observing actions within BCI feedback results in the development of mu/beta ERD, with a temporal profile similar to that observed during simple action observation. This finding suggests that the sensorimotor areas involved in “action mirroring” are also activated during BCI feedback analysis. Furthermore, significant mu range ERD was observed during anticipation of BCI feedback, indicating that feedback anticipation and prediction in our paradigm involve processes of action prediction similar to those involved in simple action observation anticipation (see Figures 2, 4). Notably, a large effect size was observed in the pre-feedback mu amplitude decrease: for the identified temporal cluster, the calculated Cohen’s d value was 0.9.

It was surprising that no differences in mu/beta ERD were observed between correct and error BCI outputs, even though wrong actions were less unexpected and have a lower probability than those of correct actions. This finding is in contrast to previous research that showed that mu/beta ERD are sensitive to action expectancy. At the same time, prior studies have suggested that mu ERD is more dependent on the somatosensory familiarity of the action than its semantic correctness (Calvo-Merino et al., 2006; Syrov et al., 2022). On the other hand, beta ERS, which is characterized by an amplitude increase in low-beta (12–20 Hz) over central cortical regions during action execution, imagery, and observation, is thought to be more strongly linked with highly hierarchical action estimation (Muthukumaraswamy and Johnson, 2004). An increase in beta ERS was found in error BCI output cases, i.e., when the presented feedback movement differed from what the subject had chosen (see below).

### 4.3. Error monitoring and BCI-feedback processing

#### 4.3.1. Error-related potentials

Error-related potentials are specific EEG signatures associated with detecting errors or conflicts during information processing DD (Joch et al., 2017; Glazer et al., 2018). Error-related negativity (Ne) and positivity (Pe) are two well-known types of ErrPs that reflect different aspects of error processing. Ne or ERN is related to error identification and conflict processing, and its source is believed to be the anterior cingulate cortex (ACC) (Dehaene et al., 1994; Glazer et al., 2018). Pe is a positive deflection following Ne and is related to error awareness (Hester et al., 2005; Glazer et al., 2018). Pe amplitude reflects cognitive processes associated with potential error consequences and post-error adjustments of behavior (Danielmeier and Ullsperger, 2011). Pe generators are associated with the prefrontal cortex, ACC, and insular cortex (Pezzetta et al., 2023).

It has been shown that the same type of neurophysiological reactions reflected in ErrPs can also be revealed when monitoring the errors of others. These potentials are called observed Ne (oNe) and observed Pe (oPe) (Koban and Pourtois, 2014; Pyasik et al., 2022). They are similar to Ne and Pe in terms of their distribution and location, but have smaller amplitudes and a longer latency. Specifically, oNe is registered between 50 and 350 ms, and oPe latency is closer to 600 ms after the observed motor error (Pezzetta et al., 2023).

In our study, we investigated ErrPs in response to movements presented as feedback in the P300-BCI paradigm. Our findings revealed a cluster of significant differences falling within the time range corresponding to the ERP with characteristics similar to the oPe: a latency range of 500–700 ms and amplitude peaking at the FCz channel (Pezzetta et al., 2023). However, we found no significant differences in the oNe potential. One possible explanation for this discrepancy may be that errors occurring within the context of a Brain-Computer Interaction (BCI) paradigm are processed differently. However, the authors of BB (Ferrez and Millán, 2005, 2008) discovered significant Ne in BCI errors as well as the late negative error-related component unique to brain-computer interaction. We observed a similar slow late deflection after oPe, which was slightly more negative in error feedback, but this difference was also insignificant. Another explanation could be the type of feedback presented in our study, which was movies of finger flexion, and the possibility of a time jitter when participants noted the flexion start. This could have resulted in ERPs’ peak smearing, which might hide differences (Guy et al., 2021). Moreover, we used a non-parametric permutation test instead of the commonly used approach with *a priori* channel and latency range selection, which has a higher potential to reveal false-negative results (Luck and Gaspelin, 2017).

#### 4.3.2. Beta synchronization

In the time-frequency domain, the observation of motor errors has been linked to enhanced activity in the lower subrange within sensorimotor beta activity. Here we found a significant increase in the beta ERS amplitude during the observation of error-BCI outputs. This increase occurred in the low beta subrange, with contralateral predominance in the sensorimotor areas (Figure 3).

Beta ERS is thought to reflect the inhibition of the M1 area under the activity of somatosensory and/or premotor cortices, which may be involved in action termination or the prevention of involuntary action imitation (Archibald et al., 2001). Numerous studies have demonstrated error-related beta ERS, with authors reporting increased beta power during the execution of incorrect and erroneous actions (van Driel et al., 2012; Tan et al., 2014; Torrecillos et al., 2015; Little et al., 2019) or the performance of semantically meaningless actions (Van Elk et al., 2010b).

Moreover, beta ERS has also been observed to increase in individuals who observe erroneous actions performed by others, highlighting the role of beta ERS-generating networks as part of the action observation network (AON) (Koelewijn et al., 2008; Pezzetta et al., 2023). Notably, Honaga et al. (2010) have demonstrated a decrease in beta ERS in individuals with autism spectrum disorder during action observation, which further supports the involvement of beta ERS in the AON.

The observed increase in beta activity during the observation of erroneous movements may be associated with inhibition of the motor cortex, which could be due to the termination or correction of internally imitated actions. This phenomenon is similar to beta ERS during real movement errors, which is thought to reflect inhibition of the current action (Stapel et al., 2010; Walsh et al., 2010). Although beta ERS is commonly thought to reflect the processing of action errors in relation to their meaning, in this study we observed beta ERS in response to simple intransitive (meaningless) actions that were presented as BCI feedback. At the same time, these observed actions served to signal users about their BCI performance, which was further confirmed by the presence of oErrPs (see Figure 5). Based on these findings, it is possible to hypothesize that knowledge regarding the correctness of BCI feedback may be represented directly in the motor system (Van Elk et al., 2010a).

### 4.4. Future applications and limitations of the study results

It has been demonstrated that the function of action monitoring networks is impaired in stroke patients. It is reflected in a mu/beta ERD decrease and a decrease in oPe amplitude. Training of these networks via action observation therapy is suggested as a promising tool for motor rehabilitation. Activation of the mirror neuron system in post-stroke patients has already shown its therapeutic effect. It has been demonstrated that patients who have had a stroke have a decrease in the function of their action monitoring networks. This decline is reflected in a decrease in AO-related mu/beta ERD and oPe amplitude (Frenkel-Toledo et al., 2014; Pyasik et al., 2022). Therefore, action observation therapy is being proposed as a promising tool for motor rehabilitation to train these networks (Ertelt et al., 2007). The therapeutic benefits of activating the mirror neuron system in post-stroke patients have already been demonstrated. Studies (Sun et al., 2016; Tani et al., 2018) have demonstrated improvements in both motor skills and function of the action observation network (AON) following action observation therapy. They have also recommended the use of EEG markers of AON activation to assess the rehabilitation progress. AO therapy may have greater benefits compared to motor imagery therapy, because some patients with motor impairments may not be able to successfully engage in motor imagery tasks, leading to insufficient activation of the relevant brain regions (Braun et al., 2006).

While AO therapy may be effective, it is a passive procedure that may not engage patients, leading to reduced motivation and insufficient activation of action monitoring mechanisms. To address this issue, we propose the use of a BCI paradigm with AO feedback. Cases of the coupling of BCI technologies with action observation have already been the subject of several studies (Neuper et al., 2009; Kim T. et al., 2016; Choi et al., 2019). Our approach is unique in that we use AO-feedback inside the P300-based BCI, making it easier to use for post-stroke patients (Piccione et al., 2006; Guger et al., 2009).

In addition, based on our results, we can suggest that providing the highest possible BCI accuracy may not be necessary, as even erroneous BCI outputs can still activate sensorimotor contours within the action monitoring system. We believe that observing erroneous actions within the BCI loop may even be beneficial, as it engages mechanisms for monitoring movement errors. Our approach also avoids the need to estimate observed action goals, which may be difficult for post-stroke patients, instead focusing on choosing the target finger to move and comparing the BCI-launched movement with the desired one.

Finally, we would like to highlight several limitations of our study. Our findings show that anticipating action observation causes pre-movement event-related desynchronization (ERD) in the alpha range, which we interpret as activation of sensorimotor cortical areas. However, we acknowledge that the presentation of the cross and consequent action anticipation may also engage visual attention and lead to occipital alpha-ERD contamination of mu ERD signals (Bollimunta et al., 2011; Bazanova and Vernon, 2014). To address this potential issue and ensure that all mu ERD were analyzed, we used the common spatial pattern (CSP) method to separate specific spatial sources and only used signals from sources with centrally distributed patterns (see Figures 2, 3).

Moreover, we hypothesized that we would observe EEG correlates of BCI feedback anticipation, including feedback-related negativity and stimulus-preceding negativity, both of which are typically observed prior to the presentation of feedback stimuli (Glazer et al., 2018). However, our results did not reveal any such activity. One possible explanation for this finding could be related to our selection of the baseline correction interval in the ERP analysis and/or the relatively small number of averaged epochs.

## 5. Conclusion

We found that during the anticipation period of the action in the passive observation paradigm, there was a significant desynchronization in mu oscillations, but not in beta, whereas during the observation period of the action itself, there was a gradual decrease in amplitude in both mu and beta bands. Similar results were obtained in the active paradigm of AO, where participants observed actions presented as feedback in a P300-based BCI loop. These data suggest activation of somatosensory cortical regions during observation of BCI feedback. However, the correctness of the observed feedback actions did not affect the amplitude of the mu/beta ERD. At the same time, the synchronization of EEG activity in the low-beta range was significantly higher during the incorrect feedback observation. Error-related potentials indicate that processes of error monitoring and analysis took place during feedback observation. This suggests that sensorimotor areas involved in the action monitoring system are engaged in BCI feedback estimation. Our results confirm that active action monitoring within the P300 BCI loop is a potentially helpful technique for post-stroke rehabilitation of the motor system, as it engages the brain’s action monitoring and action observation networks.

## Data availability statement

The raw data supporting the conclusions of this article will be made available by the authors, without undue reservation.

## Ethics statement

The studies involving human participants were reviewed and approved by the Lomonosov Moscow State University Committee for Bioethics. The patients/participants provided their written informed consent to participate in this study.

## Author contributions

NS and AK conceived the idea, developed the theory, and designed the experiment. NS and AM performed the experiment. NS and LY processed the data and drafted the manuscript. AK supervised the project. All authors discussed the results and contributed to the final manuscript.
